# Effects of annual *vs* triennial mammography interval on breast cancer incidence and mortality in ages 40–49 in Finland

**DOI:** 10.1038/bjc.2011.372

**Published:** 2011-09-20

**Authors:** I Parvinen, S Chiu, L Pylkkänen, P Klemi, P Immonen-Räihä, L Kauhava, N Malila, M Hakama

**Affiliations:** 1 University of Turku, Turku, Finland; 2 School of Health Sciences, University of Tampere, Tampere, Finland; 3 Finnish Cancer Registry, Pieni Roobertinkatu 9, FI-00130 Helsinki, Finland; 4 Department and Graduate Institute of Health Care Management, Chang Gung University, Kwei-Shan, Taiwan; 5 Turku University Hospital, General Practice, Turku, Finland; 6 Hospital District of Southwest Finland, Turku, Finland

**Keywords:** screening, breast cancer, mortality, population-based

## Abstract

**Background:**

The aim of this study was to evaluate the effects of mammography screening invitation interval on breast cancer mortality in women aged 40–49 years.

**Methods:**

Since 1987 in Turku, Finland, women aged 40–49 years and born in even calendar years were invited for mammography screening annually and those born in odd years triennially. The female cohorts born during 1945–1955 were followed for up to 10 years for incident breast cancers and thereafter for an additional 3 years for mortality.

**Results:**

Among 14 765 women free of breast cancer at age 40, there were 207 incident primary invasive breast cancers diagnosed before the age of 50. Of these, 36 women died of breast cancer. The mean follow-up time for cancer incidence was 9.8 years and for mortality 12.8 years. The incidence of breast cancer was similar in the annual and triennial invitation groups (RR: 0.98, 95% confidence interval (CI): 0.75–1.29). Further, there were no significant differences in overall mortality (RR: 1.20, 95% CI: 0.99–1.46) or in incidence-based breast cancer mortality (RR: 1.14, 95% CI: 0.59–1.27) between the annual and triennial invitation groups.

**Conclusions:**

There were no differences in the incidence of breast cancer or incidence-based breast cancer mortality between the women who were invited for screening annually or triennially.

Even though breast cancer screening in women aged over 50 years is a well-accepted practice in many countries, and controlled randomised trials have shown mortality reduction of about 25% (IARC Handbook of Cancer Prevention, 2002) in women aged 50–69 years, there is still some debate on screening in this age group. In Finland, the results of the mammography screening programme are in line with those of other countries with an effect of somewhat >20% (Hakama *et al*, 1997; Sarkeala *et al*, 2008).

In women younger than 50 years, the benefit of mammography screening is considered to be less clear. Even though a meta-analysis of previous randomised trials showed a 15% mortality reduction in women invited at age 40–49 years at entry (Smith *et al*, 2004), this finding could be due in part to the screening of these women after the age of 50 years. The results from the most recent randomised controlled trial, the ‘Age’ trial in the United Kingdom, are in line with the 2004 meta-analysis results, showing a 17% reduction in breast cancer mortality in the intervention group invited for screening annually (relative risk of 0.83, 95% confidence interval (CI): 0.66–1.04), and a 24% mortality reduction (RR of 0.76, with 95% CI: 0.51–1.01) in those actually screened – that is, with adjustment for non-compliance (Moss *et al*, 2006). Thus, the evidence from the long-term follow-up suggests that this younger age group too benefits from screening.

A recent analysis of the WHO database of breast cancer mortality trends in 30 European countries showed a 19% reduction in age-adjusted breast cancer mortality in Europe from 1989 to 2006 (Autier *et al*, 2010). The greatest mortality reduction, 37%, was observed among women under 50 years of age, and it was seen also in countries where screening at that age is uncommon. The main contributors to this observed breast cancer mortality reduction are considered to be treatment, screening, and system efficiency (Autier *et al*, 2010). At present, there are no consistent guidelines for mammography screening of women aged 40–49 years. In previous randomised studies, various screening invitation intervals, ranging from 12 to 24 months, have been used (Smith *et al*, 2004; Moss *et al*, 2006). The studies conclude that, if anything, the interval should be short rather than long compared with the commonly used 24 months in women aged 50–69 years. However, no consensus exists, which interval, if any, should be used in young age groups. Therefore, more data are needed on screening intervals at ages 40–49 years.

Finland started a biannual nationwide breast cancer screening programme for women aged 50–59 in 1987 and extended the screening to women aged 60–69 in 2007. However, the city of Turku, in southwest Finland, with a population of about 170 000, took the screening further in 1987 by also inviting women aged 40–49 years annually (even year-of-birth cohorts) or triennially (odd birth-year cohorts) and women aged 70–74 biannually. Results of the screening among elderly women were published previously (Parvinen *et al*, 2006). Preliminary results concerning the screening effects based on breast cancer survival (screened *vs* non-screened) have also been reported (Klemi *et al*, 2003). The purpose of the present study is to compare the effect of the screening invitation every year to invitation every third year on incidence-based breast cancer mortality in women invited to screening at the age of 40–49 years.

## Materials and methods

### Screening invitation schedule and fixed cohort analyses

**Figure 1:**
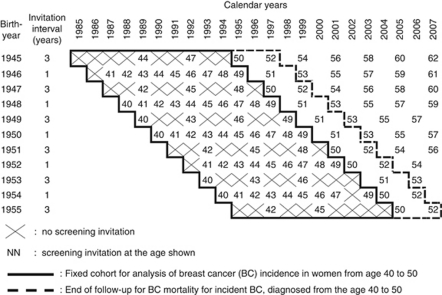
Study design of mammography with annual *vs* triennial screening interval among women aged 40–49 in 1985–2007 in Turku, Finland.

Mammography screening, as part of the nationwide screening programme in Finland, started in 1987 in the city of Turku. In addition to the nationwide screening programme, women aged 40–49 were invited to screening with a personal letter and giving a pre-fixed appointment time according to the scheme described in Figure 1. All women irrespective of their previous cancer history or familial background were invited for screening. Two-view, double-read mammograms were taken in one screening centre in Turku where altogether eight radiologists were involved in the reading process throughout the study period. The attendance rate was 85% and the recall rate was 3.5% in 1987–2003.

Women were assigned by year of birth into two groups; women born in even calendar years were invited for screening every year, while those born in odd calendar years were invited every 3 years. Further, in the present study, we balanced the two arms by age. The follow-up started at the age of 40 (irrespective of whether one was actually invited for screening or not) and ended at age 49 (for incident breast cancer cases) or at age 52 (for incidence-based breast cancer mortality and all-cause mortality). Any effects of differing calendar time were balanced by restriction of the calendar years of invitation to yield the same median calendar year in both arms.

The material was analysed based on the intention-to-screen principle; that is, the arms were compared by the status of invitation, not by actual screening. Only invasive breast cancers that were diagnosed from age 40 to age 49 years and deaths due to these breast cancers up to age 52 years were included. We call this outcome as the incidence-based mortality, also referred to as refined mortality.

In total, five 1-year birth cohorts of women born in even years from 1946 to 1954 were invited annually, and six birth cohorts of women born in odd years from 1945 to 1955 were invited triennially at the age of 40–49 years (Figure 1). The number of women in each 1-year birth cohort varied from 1120 to 1526.

The analyses were performed according to the intention-to-screen principle with the specific aim of comparing intensive (annual) and less intensive (triennial) invitation groups.

### Registry data

The new primary breast cancers were found by linking the study cohort with the Finnish Cancer Registry (FCR) database, and these data formed the breast cancer database for the study. The Turku clinical breast cancer database included comprehensive information on follow-up for all women diagnosed with breast cancer from the first invitation to the end of follow-up (up to 31 December 2007) or death, whichever occurred first. The FCR database is a comprehensive population-based nationwide database (see http://www.cancer.fi and Teppo *et al*, 1994).

The data on breast cancer cases and deaths were further validated; all primary invasive breast cancers diagnosed during the study period were cross-checked case by case between the FCR and the Turku breast cancer database. All mismatching cases were resolved by means of detailed patient medical record information.

For the analysis of overall mortality, the study cohort was linked with the database of Statistics Finland to obtain the number and causes of all deaths in this population.

The deaths from breast cancer in the Statistics Finland database were all breast cancer deaths (*n*=50) in the study cohort (also including deaths from breast cancers diagnosed before the entry to the study cohort – that is, prevalent breast cancers). In the incidence-based mortality analyses, we included only breast cancer deaths of women diagnosed with breast cancer during the study period (i.e., incident breast cancers at ages 40–49) (*n*=36).

The study was approved by the Ethics committee of the Hospital District of Southwest Finland and the investigators also received authorisation for the use of data from the Ministry of Social Affairs and Health.

### Statistical analyses

The primary outcome of this study was breast cancer death in women diagnosed with breast cancer between the ages of 40 and 49 years. The follow-up lasted 3 years after the final possible invitation for screening at the age of 49 years – that is, until the end of the year in which the women reached 52 years of age. For the calculation of exact person-years, follow-up for each individual woman started on the day when she turned 40 and ended on the last day when she was 49 years old (for breast cancer incidence) or 52 years (for mortality analyses).

For those women who died or emigrated outside the country before they turned 50, the last day of follow-up was the date of death or date of emigration.

For analysis of breast cancer mortality between women invited annually and triennially, a Poisson regression model was applied to estimate the relative rate and its 95% CI. All statistical analyses were conducted by means of SAS software (version 9.1; SAS Institute, Inc., Cary, NC, USA, 2007).

## Results

In all, 14 765 women were included in the analyses after excluding 43 women because of prevalent breast cancer. Of these women, 7839 were in the triennial invitation group and 6926 in the annual group. In total, 77 083 and 68 018 person-years of follow-up were available for breast cancer incidence (Table 1) and 100 508 and 88 543 person-years in the analyses for incidence-based breast cancer mortality in the triennial and annual invitation groups, respectively (Table 2). In all-cause mortality, the follow-up time was slightly longer in both groups since prevalent breast cancer cases were included. The overall mean follow-up time for breast cancer incidence was 9.8 years and that for incidence-based breast cancer mortality was 12.8 years.

The annually invited cohort had an average of 9.2 invitations per woman, and that of the triennial cohort had 2.8 invitations per woman. Therefore, the actual screening invitation algorithm was over three times (9.2/2.8) more intensive in the annual invitation group than in the triennial group.

In total, 207 women were diagnosed with primary invasive breast cancer. There was no difference in the incidence of breast cancer between the cohorts (RR: 0.98, 95% CI: 0.75–1.29 annual *vs* triennial group) (Table 1).

In total, 399 women in the whole cohort of 14 808 women died during the study period. All-cause mortality was 20% higher in the annual invitation group (RR: 1.20, 95% CI: 0.99–1.46) than in the triennial group. Out of those women with an incident breast cancer diagnosed (207 women), 36 died from breast cancer during follow-up. No significant difference in incidence-based breast cancer mortality was observed in women invited annually as compared with those invited triennially (RR: 1.14, 95% CI: 0.59–1.27) (Table 2).

When causes of death were analysed in more detail, no clear reason for the difference in overall mortality could be identified. A slight excess in mortality from cancers other than breast cancer was observed among those invited annually, but no reason for this excess (e.g., radiation-induced cancers) could be identified. In the triennial group, there were more violent deaths than in the annual group (see Table 3).

## Discussion

The purpose of our study was to compare the effect of the screening policy with annual and triennial invitation intervals on incidence-based (refined) breast cancer mortality. There was no evidence of a differential effect in this outcome between the groups. The women in this study were first allocated into two groups; those born in even years were invited for screening every year, and those born in odd years were invited every third year. The rationale for using fixed cohorts was to equalise the intensive (annual) and less intensive (triennial) screening arms, to enable comparison of incidence-based (refined) breast cancer mortality and all-cause mortality rates between the groups. Because the median calendar time and age were the same in both groups, there was no confounding by the increasing background risk by age and by calendar time, and the design corresponds to that of a randomised study. The use of the nationwide and population-based registry data, the validation of the registry data with the clinical database, and the comprehensive follow-up of a relatively large number of women are further clear advantages of this study.

Our study was designed according to the intention-to-screen principle, allowing evaluation of how effective the screening policies were compared with each other. Such results have implications on the practical application of screening, and on the public health policy. Therefore, the results are not influenced by contamination in the triennial arm or non-attendance in the annual screening arm. Specifically, contamination and non-attendance are inbuilt characteristics of any policy and the comparison should allow for both.

Our results are consistent with no effect of the annual schedule compared with the triennial one. Because of the lack of a control group with no screening, we cannot determine whether this result was due to a reduction in efficacy of mammography screening in this age group or whether the effectiveness of triennial screening is similar to that of annual screening. Breast cancers in younger women are considered to be more aggressive, and a more intensive screening policy for these women has been suggested for a long time (Tabár *et al*, 1987; Venta and Goodhartz, 1996). A short screening interval was also proposed because the sensitivity of mammography screening is lower in women aged 40–49 years (Bailey *et al*, 2010).

In previous randomised studies, various screening invitation intervals, ranging from 12 to 24 months, have been used (Smith *et al*, 2004; Moss *et al*, 2006). The results with Markov-chain models of breast tumour progression to determine the optimal screening interval with the data from the Swedish trials suggest that the screening interval is critical for women aged 40–49 but less so for older women (Duffy *et al*, 1997). Along the same lines, proceeding from results of randomised controlled trials, Tabar *et al* (1989) have proposed that the screening interval should be no more than 18 months for women aged 40–49 years. Consequently, for women aged 40–49, a 3-year mammography screening interval has been modelled to result in only a small, four per cent, reduction in mortality (Duffy *et al*, 1997).

The invitation design that we implemented (see Figure 1) resulted in a substantial contrast in the median number of invitations. Previous studies (Tabar *et al*, 1987, 1989; Venta and Goodhartz, 1996; Duffy *et al*, 1997; Smith *et al*, 2004; Moss *et al*, 2006) indicate that the possibility of equal effectiveness of a screening algorithm with 2.8 invitations and with 9.2 invitations between ages 40 and 49 years is not credible. The possibility remains that the programme provided only a marginal effect overall at most. This option highlights the problems in the application of research-based results to routine practice. Therefore, there is a need also to evaluate any routine application of screening services.

Death from breast cancer may take place decades after diagnosis. Therefore, it is possible that a mortality difference will appear after extended follow-up. Because the cohorts in our study were subjected to the same screening algorithm after the age of 50 years with a 2-year interval, prolonged follow-up may disclose the long-term effect in women screened before age 50. The present study has relatively low power, on account of the small numbers, and the power would also be improved with an increase in the person-years at risk.

There was a slight excess in deaths from other natural causes, including other malignancies than breast cancer (Table 3). On the other hand, an excess of violent deaths was observed in the triennially invited population. The analysis of the specific causes of deaths did not, however, provide any obvious explanations for these differences. For example, no excess in possibly radiation-induced cancers with a short lag (e.g., haematopoietic malignancies) could be observed. Therefore, the differences seen in cause-specific deaths are most likely due to chance.

In conclusion, there was no evidence of a difference in incidence-based (refined) mortality from breast cancer between the annual and triennial screening invitations under the age of 50. This is consistent with a rather similar effect (including no effect) between annual and triennial screening algorithms. Hence, our result is not proof of ineffectiveness of screening with mammography in women under 50 years in general, but points to the need for evaluating also the routine application of screening services.

## Figures and Tables

**Table 1:** The incidence (per 100 000 person-years) of breast cancer (BC) by screening invitation interval

	**Triennial**	**Annual**
Women *(n)*	7839	6926
Person-years	77 083	68 018
BC cases (*n*)	111	96
Incidence of BC (per 100 000)	144.0	141.1
RR (95% CI)	Reference	0.98 (0.75, 1.29)

Abbreviations: CI=confidence interval; RR=relative risk.

The study with mammography in 1985–2007 in Turku, Finland.

**Table 2:** All-cause mortality (per 100 000 person-years) and incidence-based^a^ breast cancer (BC) mortality by screening invitation interval

	**Triennial**	**Annual**
Person-years for all-cause mortality	100 738	88 780
Number of deaths (*n*)	194	205
Total mortality rate (per 100 000)	192.6	230.9
RR (95% CI)	Reference	1.20 (0.99, 1.46)
Person-years for incidence-based breast cancer mortality^a^	100 508	88 543
Number of BC deaths (*n*)	18	18
BC mortality (per 100 000)	17.9	20.3
RR (95% CI)	Reference	1.14 (0.59, 1.27)

Abbreviations: CI=confidence interval; RR=relative risk.

a Only deaths from incident breast cancer diagnosed at ages 40–49 years included.

The study with mammography in 1985–2007 in Turku, Finland.

**Table 3:** Number of deaths by cause and screening invitation interval

**Causes of death**	**Triennial**	**Annual**
Total number of deaths	194	205
Breast cancer deaths^a^	26	24
Other cancer deaths (BC excluded)	43	53
Oesophagus, ventricle	9	4
Colon, rectum, small intestine	3	9
Pancreas, liver	4	5
Lung	6	11
Sarcoma	1	2
Melanoma	0	2
Gynaecological	7	5
Urinary tract	3	4
Brain	4	3
Lymphoma, leukaemia, aplastic anaemia	5	5
Other (unknown primary)	1	3
Other natural causes of death	76	94
Violent causes of death^b^	49	34

a Breast cancer (BC) cases from Statistics Finland, including also deaths from BC (and other diseases) diagnosed before the start of follow-up.

b Including accidents, intoxication, suicide, and murder.

The study with mammography in 1985–2007 in Turku, Finland.
